# Mental Health Literacy of Non-mental Health Nurses: A Mental Health Survey in Four General Hospitals in Hunan Province, China

**DOI:** 10.3389/fpsyt.2020.507969

**Published:** 2020-10-27

**Authors:** Yuzhu Hao, Qiuxia Wu, Xiaoyang Luo, Shubao Chen, Chang Qi, Jiang Long, Yifan Xiong, Yanhui Liao, Tieqiao Liu

**Affiliations:** ^1^Department of Psychiatry, The Second Xiangya Hospital, Central South University, The China National Clinical Research Center for Mental Health Disorders, Chinese National Technology Institute of Psychiatry, Key Laboratory of Psychiatry and Mental Health of Hunan Province, Changsha, China; ^2^Female Psychiatric Ward, The First Psychiatric Hospital of Hengyang City, Hengyang, China; ^3^Zhejiang Provincial People's Hospital, Hangzhou, China; ^4^Shanghai Mental Health Center, Shanghai Jiao Tong University School of Medicine, Shanghai, China; ^5^Brain Hospital of Hunan Province, Changsha, China; ^6^Department of Psychiatry, Sir Run Run Shaw Hospital, School of Medicine, Zhejiang University, Hangzhou, China

**Keywords:** mental health literacy, acknowledgment, non-mental health nurse, China, mental disorder

## Abstract

**Background:** The high numbers of patients with mental illness, especially those who are misdiagnosed or delayed in treatment in China, have imposed a huge burden on the country and the society. This study was designed to investigate the mental health literacy (MHL) of non-mental health nurses.

**Method:** A cross-sectional survey was designed, and a convenient cluster sampling method was applied. We presented evidence on 601 nurses from the non-mental health department in four hospitals in Hengyang city, China. One-third of the vignettes were diagnosed with schizophrenia, depression, or generalized anxiety disorder (GAD).

**Result:** The correct identification rates for schizophrenia, depression, and GAD vignettes reached 38.9, 56.2, and 17.5%, respectively. The majority of the participants deemed that the person in each vignette needed professional help, and most of them preferred professional medical help and lifestyle interventions. As for the likely outcome for the persons described in each vignette, more than half of the participants thought that with professional help, the patients would make a full recovery, but problems would probably recur.

**Conclusion:** The MHL of non-mental health nurses is insufficient in China. This condition is particularly applicable in the case of GAD. Thus, the country must implement more mental health education programs to improve the MHL of non-mental health nurses.

## Introduction

Mental disorders are common around the whole world and burdensome for individuals and the society (1). Mental disorders have become more common across China in the past 30 years. China has a population of about 1.4 billion, and the lifetime prevalence of mental disorders (excluding dementia) accounts for 16.6% (2). Globally, 17% of the burden caused by mental, neurological, and substance use disorder was suffered by Chinese people in 2013 and accounted for 36 million global disability-adjusted life years (10% for all disease burden) (3). Mental disorders are associated with enormous economic burden, including the high cost of treatment and regression of the society. In China, the total costs of mental disorders accounted for more than 15% of total health expenditure in 2013 and 1.1% of China's gross domestic products (4). Although continuous efforts have been made by the Chinese government, the mental health resources available in China remain limited. In 2015, the numbers of mental health institutes and psychiatric beds reached 1,650 and 228,000, respectively. A total of 1.49 psychiatrists are available per 100,000 people in China, whereas 2.03 psychiatrists are accessible in middle- and high-income countries worldwide (5). Given that specific mental disorders are mainly manifested as physical discomfort [such as anxiety disorder which is often accompanied by autonomic symptoms, including headache, palpitations, tightness in the chest, and mild stomach (6)], many patients seek help in internal medicine departments, such as those for neurology, gastroenterology, and cardiology. A total of 44.7, 25.7, and 7.9% of people with anxiety, substance, and mood disorders, respectively, had/have made psychotherapy contact (7). Long waiting time before treatment contact might cause the delayed treatment of diseases. Previous studies have demonstrated that delay time ranges from 6 to 8 years for mood disorders and 9 to 23 years for anxiety disorders without normal treatment contact (8). The long duration of untreated mental illness also results in poor treatment outcomes (9).

Mental health literacy (MHL) was first put forward by Jorm et al. in 1997 (10). This concept was defined as the knowledge and beliefs about mental disorders, aiding in the recognition, management, and prevention of such conditions. MHL includes the following components: the ability to recognize specific disorders; knowing how to seek mental health information; knowledge of risk factors and causes; knowledge of self-treatments; knowledge of professional help available; attitudes that promote recognition and appropriate help-seeking. Theoretically, improving MHL could improve the rates of early identification and help-seeking for mental illness, increase early intervention and successful treatment, and reduce the chances of increasing severity of disorders.

In the Chinese mental healthcare system, doctors and nurses usually work collaboratively to build a patient-centered care environment. Nurses are medical staff members who are in direct contact with patients and their families. Being in direct and long contact with patients, nurses have more opportunities to observe them. The initial identification of mental disorders from nurses helps doctors to further understand and deal with patients' conditions and improve the early identification and referral rates. Although a series of studies for MHL has been conducted among the public and professionals (11–15), a limited number of research used vignettes as a tool for studying non-mental health nurses. This study aimed to evaluate the identification and beliefs of Chinese non-mental health nurses about the interventions and outcomes regarding three common mental disorders, namely, schizophrenia, generalized anxiety disorder (GAD), and depression.

## Methods

### Sample

We used a cross-sectional survey and followed a cluster convenience sampling method. The survey was conducted between 2014 and 2015 in four general hospitals located in Hengyang, which is one of the prefecture-level cities in Hunan province, China. These hospitals are the First Affiliated Hospital of the University of South China, the First People's Hospital of Hengyang, the Fifth People's Hospital of Hengyang, and the People's Hospital of Hengyang County, which are tertiary or secondary hospitals. All these hospitals are teaching hospitals. In addition to mental health nurses, each hospital has nearly 400 nurses for the other departments. Thus, the total sample size was 1,600. We recruited nurses who are working in non-mental health departments to complete the questionnaire anonymously. We distributed 610 questionnaires, with 601 non-mental health nurses successfully completing the questionnaires. The response rate was 98.5%. The numbers of responses for each of the three vignettes types were as follows: schizophrenia, *n* = 198; depression, *n* = 203; GAD, *n* = 200.

The research program was approved by the ethics committee of the Second Xiangya Hospital of Central South University. Before completing the questionnaires, we stated the aim of the study and requested consent from the participants to participate by filling in the questionnaire.

### Survey Questionnaires

The instrument used for data collection was adapted from that used by Jorm et al. (10) to investigate MHL among Australian samples. After obtaining the permission of the original author, Dr. Liu Wei translated and modified the questionnaires to conform to Chinese conditions. Dr. Liu removed several interventions which were used in the Australian samples but are inapplicable for Chinese mental services and included additional resources and treatment options, such as having a massage, Qigong/Tai Chi therapy, and acupuncture, to fit with the Chinese medical practices and beliefs (11). Dr. Liu Wei also examined the validity and reliability of the questionnaire (11).

First, demographic information (including age, gender, marital status, education level, work duration, and hospital level) of the participants were collected. Second, the participants were randomly assigned to one of the three vignettes describing GAD, depression, or schizophrenia. Each vignette was designed to satisfy the diagnostic criteria for schizophrenia, depression, or GAD in accordance with the *Diagnostic and Statistical Manual of Mental Disorders* (4th edition) and *International Statistical Classification of Diseases and Related Health Problems* (10th revision). Following the vignettes, the participants were asked to select one “most likely” diagnosis from a series of diagnostic options. Only one choice should be given to all questions. The participants were also requested to answer how the person in the vignette would be best helped, and they were allowed to select three answers. Afterward, the participants were asked to rate the likely effect of a range of interventions (rated as “helpful,” “harmful,” or “neither”) to assess their beliefs about the treatments. Choices about the possible results with or without professional help in the vignettes were also collected. Details of the questionnaire and the vignettes are presented below.

### Statistical Analysis

The data were entered by double-entry strategy in EpiData version 3.1 (EpiData Association, Odense, Funen, Denmark). All statistical analyses were conducted with IBM-SPSS 25.0 software (IBM Corp., Armonk, NY, USA). In this survey, descriptive analyses using median (interquartile range) for skewed variables and frequency (percentage) for categorical variables were performed. Then, we computed the percentage and 95% confidence interval (CI) for helpful interventions and the best method of help. We use the Chi-square test to investigate whether any significant difference exists in each vignette in terms of the identification rate and calculated odd rate (OR) and 95% CI. In each vignette, general demographic data were compared between those who had been correctly identified and those who had not. For each item in helpful intervention and best method of help, we also used Chi-square test to study the differences between different vignettes in the proportion of helpfulness.

## Results

No significant difference was observed among the participants for the three vignettes in terms of sex, age, marital status, educational level, and work duration. All participants were female nurses from comprehensive departments. As shown in Table 1, the median age of all participants was 31 years old. About 61.7% of the participants are married. About 27.3% of the participants finished a bachelor's degree, and the others completed a junior college degree or secondary specialized degree. Medium work duration was 9.5 years, and about half of the participants worked in a tertiary hospital.

**Table 1:** Socio-demographic characteristics of participants by vignette.

**Participants characteristics**	**GAD vignette**		**Schizophrenia**		**Depression vignette**		***P*-value**
	**(*****n*** **=** **200)**		**vignette (*****n*** **=** **198)**		**(*****n*** **=** **203)**		
	***n***	**%**	***n***	**%**	***n***	**%**	
**Sex**
Female	200	100.0	198	100.0	203	100.0	
Age (years)^*^	31.0	25.0–37.0	30.5	25.0–37.0	31.0	25.0–37.0	0.989
**Marital status**
Married	124	62.0	121	61.2	126	62.1	0.996
Single	70	35.0	72	36.4	71	35.0	
Other status	6	3.0	5	2.5	6	3.0	
**Educational level**
Secondary specialized degree	34	17.0	33	16.7	32	15.8	0.996
Junior college degree	112	56.0	110	55.6	116	57.1	
Bachelor's degree	54	27.0	55	27.8	55	27.1	
Work duration (years)^*^	9.50	4.0–16.0	9.0	4.0–15.0	10.0	3.0–16.0	0.994
**Hospital level**
Tertiary hospital	97	48.5	98	49.5	102	50.2	0.940
Secondary hospital	103	51.5	100	50.5	101	49.8	

* *Data descriptive with median (interquartile range)*.

As for the diagnosis, Table 2 shows the frequency of participants who labeled all problems in each vignette. In the depression vignette, 56.2% (*n* = 114) of the non-mental health registered nurses provided correct answers. The correct identification rate for the schizophrenia vignette was 38.9% (*n* = 77), which was lower than the rate for the depression vignette. A total of 17.5% (*n* = 35) of the participants correctly identified the problems for the GAD vignette. We compared the correct identification rate among these three vignettes. The result shows that compared with GAD, schizophrenia was more likely to be identified correctly (OR: 3.00; CI: [1.89, 4.77]; *P* < 0.001). Depression was also considerably easier to properly identify than GAD (OR: 6.040; CI: [3.82, 9.55]; *P* < 0.001). Depression was more likely to gain better recognition than schizophrenia (OR: 2.01; CI: [1.35, 3.00]; *P* < 0.001).

**Table 2:** Frequency of participants who labeled each problem shown in the vignettes.

**Disorders**	**GAD vignette**		**Schizophrenia**		**Depression vignette**	
	**(*****n*** **=** **200)**		**vignette (*****n*** **=** **198)**		**(*****n*** **=** **203)**	
	***n***	**%**	***n***	**%**	***n***	**%**
Stress	28	14.0	8	4.0	14	6.9
Depression	13	6.5	13	6.6	**114**	**56.2**
Schizophrenia	4	2.0	**77**	**38.9**	1	0.5
Psychological problems	8	4.0	7	3.5	10	4.9
Mental illness	7	3.5	42	21.2	15	7.4
Physical illness	5	2.5	2	1.0	7	3.4
Emotional problem	9	4.5	2	1.0	17	8.4
Self-esteem	4	2.0	0	0	0	0
Spirit intrusion	1	0.5	0	0	0	0
Phobia	10	5.0	10	5.1	0	0
Social phobia	18	9.0	9	4.5	4	2.0
GAD	**35**	**17.5**	4	2.0	3	1.5
PTSD	2	1.0	5	2.5	4	2.0
Acute anxiety attack	16	8.0	2	1.0	6	3.0
OCD	20	10.0	5	2.5	0	0
Personality disorder	8	4.0	6	3.0	0	0
Caused by physical illness	5	2.5	4	2.0	7	3.4
Other	2	1.0	0	0	0	0
Do not know	5	2.5	2	1.0	1	0.5

*GAD, generalized anxiety disorder; OCD, obsessive-compulsive disorder; PTSD, post-traumatic stress disorder. The bold value means the correct identification rate in each vignettes*.

In the GAD vignette, a significant difference (*P* < 0.05) in the work duration was observed between those who recognized correctly the condition and those who did not. A significant difference was observed in the education level between those who recognized depression in the vignette correctly and those who did not (*P* < 0.05). No significant difference was found among the participants who recognized correctly or not schizophrenia.

Tables 3, 4 show the non-mental health nurses' attitudes toward treatment for all the vignettes. Most of the participants thought they needed professional help in diagnosing GAD (85.5%), depression (89.2%), and schizophrenia (94.9%).

**Table 3:** Frequency of participants who answered whether professional help is needed in diagnosing the vignettes.

**Answers**	**GAD vignette**		**Schizophrenia**		**Depression vignette**	
	**(*****n*** **=** **200)**		**vignette (*****n*** **=** **198)**		**(*****n*** **=** **203)**	
	***n***	**%**	***n***	**%**	***n***	**%**
Yes	171	85.5	188	94.9	181	89.2
No	8	4.0	2	1.0	8	3.9
Do not know	21	10.5	8	4.0	14	6.9

**Table 4:** Frequency of participants who labeled each problem shown in the vignettes.

**Interventions**	**GAD vignette**		**Schizophrenia**		**Depression vignette**		***P*-value**
	**(*****n*** **=** **200)**		**vignette (*****n*** **=** **198)**		**(*****n*** **=** **203)**		
	***n***	**%**	***n***	**%**	***n***	**%**	
**People who can help**						
A typical general practitioner or family doctor	121	60.5 (53.3, 67.3)	127	64.1 (57.0, 70.7)	117	57.6 (50.5, 64.5)	0.409
A pharmacist	57	28.5 (22.5, 35.4)	55	27.8 (21.8, 34.7)	57	28.1 (22.1, 34.9)	0.987
A counselor	101	50.5 (43.4, 57.6)	109	55.1 (47.8, 62.1)	97	47.8 (40.8, 54.9)	0.340
A social worker	56	28.0 (22.0, 34.9)	52	26.3 (20.4, 33.1)	49	24.2 (18.5, 30.7)	0.677
A telephone counseling service	64	32.0 (25.7, 39.0)	58	29.3 (23.2, 36.2)	62	30.5 (24.4, 37.4)	0.823
A psychiatrist	153	76.5^B^ (69.9, 82.1)	175	88.4^A^ (82.9, 92.3)	160	78.8 (72.4, 84.1)	<0.01^a^
A psychiatrist nurse	131	65.5^B^ (58.4, 72.0)	153	77.3^A^ (70.7, 82.8)	140	69.0 (62.0, 75.2)	0.030^b^
A clinical psychologist	163	81.5^B^ (75.3, 86.5)	176	88.9^A^ (83.5, 92.8)	167	82.3 (76.2, 87.1)	0.085^c^
Help from intimate family	161	80.5 (74.2, 85.6)	151	76.3 (69.6, 81.9)	148	72.9 (66.2, 78.8)	0.197
Help from intimate friends	154	77.0 (70.4, 82.5)	140	70.7 (63.8, 76.8)	145	71.4 (64.6, 77.4)	0.300
An herbalist	88	44.0^B^ (37.1, 51.2)	66	33.3^A^ (26.9, 40.4)	83	40.9 (34.1, 48.0)	0.082^d^
Dealing with the problem on his/her own	23	11.5 (7.6, 16.9)	21	10.6 (6.8, 16.0)	26	12.8 (8.7, 18.4)	0.787
Praying to buddha for help	15	7.5 (4.4, 12.3)	10	5.1 (2.6, 9.4)	15	7.4 (4.3, 12.1)	0.542
**Medication which can help**						
Vitamins and minerals	60	30.0 (23.8, 36.9)	76	38.4 (31.7, 45.6)	74	36.5 (29.9, 43.5)	0.184
Laxatives such as lactulose or senna	26	13.0 (8.8, 18.6)	20	10.1 (6.4, 15.4)	22	10.8 (7.1, 16.1)	0.637
Tonics or herbal medicines	71	35.5 (29.0, 42.6)	65	32.8 (26.4, 39.9)	70	34.5 (28.1, 41.5)	0.852
Pain relievers, such as aspirin or acetaminophen	30	15.0 (10.5, 20.9)	28	14.1 (9.8, 20.0)	25	12.3 (8.3, 17.8)	0.727
Antidepressants	87	43.5^C^ (36.6, 50.7)	88	44.4^C^ (37.5, 51.7)	151	74.4^A^^B^ (67.7, 80.1)	<0.001^e^
Antibiotics	30	15.0 (10.5, 20.9)	24	12.1 (8.1, 17.7)	33	16.3 (11.6, 22.2)	0.484
Sleeping pills	75	37.5 (30.8, 44.6)	70	35.4 (28.8, 42.5)	72	35.5 (30.0, 42.5)	0.881
Antipsychotics	81	40.5^B^ (33.7, 47.7)	162	81.8^A^^C^ (75.6, 86.8)	88	43.3^B^ (36.5, 50.5)	<0.001^f^
Tranquilizers such as diazepam	101	50.5 (43.4, 57.6)	99	50.0 (43.1, 56.9)	89	43.8 (37.0, 51.0)	0.329
Anxiolytics	124	62.0^B^^C^ (54.9, 68.7)	91	46.0^A^ (38.9, 53.2)	93	45.8^A^^A^ (38.9, 52.9)	<0.001^g^

Symbols flagging table entries denote significant differences relative to

A *GAD*;

B *Schizophrenia*;

C *Depression*.

a *: GAD vs. Schizophrenia P < 0.01, OR: 2.34, CI: [1.36, 4.03]*.

b *: GAD vs. Schizophrenia P < 0.01, OR: 1.79, CI: [1.51, 2.79]*.

c: * GAD vs. Schizophrenia P < 0.05, OR: 1.82, CI: [1.03, 3.21]*.

d *: Schizophrenia vs. GAD P < 0.05, OR: 1.57, CI: [1.05, 2.36]*.

e *: Depression vs. GAD P < 0.001, OR: 3.77, CI: [2.48, 5.75]*.

*Depression vs. Schizophrenia P < 0.001, OR: 3.63, CI: [2.38, 5.53]*.

f: * Schizophrenia vs. GAD P < 0.001, OR: 6.61, CI: [4.18, 10.46]*.

*Schizophrenia vs. Depression P < 0.001 OR: 5.88 CI: [2.73, 9.27]*.

g *: GAD vs. Depression P < 0.01, OR: 1.92, CI: [1.29, 2.86]*.

*GAD vs. Schizophrenia P < 0.01, OR: 1.93, CI: [1.30, 2.87]*.

The participants were given a list of intervention treatments which they rated depending on helpfulness. For all the vignettes, a clinical psychologist, a psychiatrist, and helping intimate family were considered the most helpful (>70%). Praying to Buddha, dealing with the problem on their own, and seeking help from a counselor were the least helpful interventions (<30%). Anxiolytics (62.0%), followed by tranquilizers (50.5%) and antidepressants (43.5%), were recognized as the most helpful by the participants for the GAD vignettes. Antipsychotics were recognized as helpful by 81.8% of the participants for the schizophrenia vignette, followed by tranquilizers (50%) and anxiolytics (46.0%). Antidepressants were recognized as helpful by 74.4% of the participants for the depression vignette, followed by anxiolytics (45.8%) and tranquilizers (43.8%).

Compared with the GAD vignette, most participants believed that psychiatrists (*P* < 0.01; OR: 2.34; CI: [1.36, 4.03]), psychiatric nurses (*P* < 0.01; OR: 1.79; CI: [1.51, 2.79]), and clinical psychologists (*P* < 0.05; OR: 1.82; CI: [1.03, 3.21]) were more helpful in the schizophrenia vignette. Dealing with problem on his/her own was thought to be more helpful in GAD vignette than in schizophrenia (*P* < 0.05; OR: 1.57; CI: [1.05, 2.36]).

As shown in Table 5, going out was thought to be helpful by a majority of the participants for the GAD (80.5%), schizophrenia (84.3%), and depression vignettes (82.8%). For the GAD and depression vignettes, electro-convulsive therapy was considered the least helpful intervention. However, occasionally consuming alcoholic drink to relax was rated as the least helpful intervention in the schizophrenia vignette. Over 80% of the participants for the schizophrenia vignette thought that being admitted to a psychiatric ward of a general hospital or a psychiatric hospital was helpful, whereas the ratios in the GAD and depression vignettes were below 70%. The rate of participants who rated “being admitted to a psychiatric ward of a general hospital” as helpful was higher for the schizophrenia vignette than the depression vignette (*P* < 0.01; OR: 1.85; CI: [1.62, 2.94]) and GAD (*P* < 0.01; OR: 2.22; CI: [1.40, 3.51]) vignettes. More participants believed that “becoming physically more active” was more helpful in schizophrenia vignette than in GAD vignette (*P* < 0.05; OR: 1.83; CI: [1.15, 2.90]). The helpful rate of “being admitted to a psychiatric hospital” was significantly higher for the schizophrenia vignette than that of depression (*P* < 0.01; OR: 2.14; CI: [1.35, 3.40]) and GAD (*P* < 0.01; OR: 2.90; CI: [1.84, 4.58]). “Undergoing electro-convulsive therapy” was also thought to be more helpful in schizophrenia than depression (*P* < 0.05; OR: 1.64; CI: [1.06, 2.54]) and GAD (*P* < 0.01; OR: 1.80; CI: [1.16, 2.81]).

**Table 5:** Frequency of participants who rated other interventions as “helpful” in the vignettes.

**Other interventions**	**GAD vignette**		**Schizophrenia**		**Depression vignette**		***P*-value**
	**(*****n*** **=** **200)**		**vignette (*****n*** **=** **198)**		**(*****n*** **=** **203)**		
	***n***	**%**	***n***	**%**	***n***	**%**	
Becoming physically more active, such as playing more sports, or doing a lot more walking or gardening	138	69.0^B^ (62.0, 75.2)	159	80.3^A^ (73.9, 85.5)	152	74.9 (68.2, 80.6)	<0.05^h^
Reading about people with similar problems and how they have dealt with them	149	74.5 (67.8, 80.3)	146	73.7 (66.9, 79.6)	145	71.4 (64.6, 77.4)	0.769
Getting out more	161	80.5 (74.2, 85.6)	167	84.3 (78.4, 89.0)	168	82.8 (76.7, 87.5)	0.597
Staying at home and resting	91	45.5^B^ (37.5, 52.7)	55	27.8^A^^C^ (21.8, 34.7)	81	39.9^B^ (33.2, 47.0)	<0.001^i^
Attending courses or relaxation, stress management, meditation, or yoga	114	57.0 (49.8, 63.9)	116	58.6 (51.4, 65.5)	120	59.1 (52.0, 65.9)	0.905
Massage to relax	122	61.0 (53.8, 67.7)	115	58.1 (50.9, 65.0)	126	62.1 (55.0, 68.7)	0.701
Cutting out alcohol altogether	118	59.0 (51.8, 65.8)	120	60.6 (53.4, 67.4)	120	59.1 (52.0, 65.9)	0.936
Having an occasional alcoholic drink to relax	49	24.5 (18.8, 31.2)	47	23.7 (18.1, 30.4)	57	28.1 (22.1, 34.9)	0.565
Qigong/Tai Chi therapy	77	38.5 (31.8, 45.7)	73	36.9 (30.2, 44.0)	81	39.3 (33.2, 47.0)	0.823
Acupuncture therapy	75	37.5 (30.8, 44.6)	71	35.9 (29.3, 43.0)	77	37.9 (31.3, 45.0)	0.903
Psychotherapy	143	71.5 (64.6, 77.5)	152	76.8 (70.1, 82.3)	153	75.4 (68.7, 81.0)	0.457
Hypnosis	106	53.0 (45.8, 60.0)	115	58.1 (50.9, 65.0)	122	60.1 (53.0, 66.8)	0.334
Aromatic therapy	89	44.5 (37.5, 51.7)	84	42.4 (35.5, 49.6)	96	47.3 (40.3, 54.3)	0.616
Being admitted to a psychiatric ward of a general hospital	131	65.5^B^ (58.4, 72.0)	160	80.8^A^^C^ (74.5, 85.9)	141	69.5^B^ (62.6, 75.6)	<0.01^j^
Being admitted to a psychiatric hospital	120	60.0^B^ (52.8, 66.8)	161	81.3^A^^C^ (75.0, 86.3)	136	67.0^B^ (60.0, 73.3)	<0.001^k^
Undergoing electro-convulsive therapy	45	22.5^B^ (17.0, 29.0)	68	34.3^A^^C^ (27.8, 41.5)	49	24.1^B^ (18.5, 30.7)	<0.05^l^
Going on a special diet or avoiding certain foods	71	35.5 (29.0, 42.6)	70	35.4 (28.8, 42.5)	81	39.9 (33.2, 47.0)	0.561

Symbols flagging table entries denote significant differences relative to

A *GAD*;

B *Schizophrenia*;

C *Depression*.

h *: Schizophrenia vs. GAD P <0.01, OR: 1.83, CI: [1.15, 2.90]*.

i *: GAD vs. Schizophrenia P < 0.001, OR: 2.34, CI: [1.36, 4.03]*.

*Schizophrenia vs. Depression P < 0.01, OR: 1.72, CI: [1.14, 2.63]*.

j *: Schizophrenia vs. GAD P < 0.001, OR: 2.22, CI: [1.40, 3.51]*1.

*Schizophrenia vs. Depression P < 0.01, OR: 1.85, CI: [1.62, 2.94]*.

k *: Schizophrenia vs. GAD P < 0.001, OR: 2.90, CI: [1.84, 4.58]*.

*Schizophrenia vs. Depression P < 0.01, OR: 2.14, CI: [1.35, 3.40]*.

l *: Schizophrenia vs. GAD P < 0.01, OR: 1.80, CI: [1.16, 2.81]*.

*Schizophrenia vs. Depression P < 0.05, OR: 2.14, CI: [1.06, 2.54]*.

We asked the participants about how they would help the person in the vignettes if they were one of their families or friends. They were allowed to select 3 answers from 23 options (Table 6). Listening/talking with a person was thought to be the most helpful in the GAD (47.0%) and depression (40.9%) vignettes. In the schizophrenia vignette, encouraging the person to seek help from a psychologist was thought to be the most helpful choice, followed by listening/talking with the person and encouraging them to consult a counselor. “Encourage the person to go to a mental health hospital” was considered more helpful in schizophrenia than in depression (*P* < 0.05; OR: 1.64; CI: [1.00, 2.67]) and GAD (*P* < 0.01; OR: 2.41; CI: [1.41, 4.12]).

**Table 6:** Frequency of participants selecting each category to answer how the person in the vignettes could be best helped.

**Help methods**	**GAD vignette**		**Schizophrenia**		**Depression vignette**		***P*-value**
	**(*****n*** **=** **200)**		**vignette (*****n*** **=** **198)**		**(*****n*** **=** **203)**		
	***n***	**%**	***n***	**%**	***n***	**%**	
Listen/talk with the person	**94**	**47.0 (40.0, 54.0)**	**76**	**38.4 (31.5, 45.2)**	**83**	**40.9 (34.1, 47.7)**	0.200
Accompany the person to professional help	55	27.5 (21.3, 33.7)	53	26.8 (20.5, 33.0)	43	21.2 (15.5, 26.9)	0.278
Contact professional help on the person's behalf	34	17.0 (11.7, 22.3)	35	17.7 (12.3, 23.0)	37	18.2 (12.9, 23.6)	0.949
Encourage the person to seek help	35	17.5 (12.2, 22.8)	44	22.2 (16.4, 28.1)	30	14.8 (9.9, 19.7)	0.148
Encourage the person to see a community physician	11	5.5 (2.3, 8.7)	6	3.0 (0.6, 5.4)	7	3.4 (0.9, 6.0)	0.402
Encourage the person to see a counselor	59	29.5 (23.1, 35.9)	48	24.2 (18.2, 30.3)	57	28.1 (21.8, 34.3)	0.476
Encourage the person to see a psychiatrist	**62**	**31.0 (24.5, 37.5)**	**59**	**29.8 (23.4, 36.2)**	**67**	**33.0 (26.5, 39.5)**	0.782
Encourage the person to see a psychologist	**65**	**32.5 (26.0, 39.0)**	**78**	**39.4 (32.5, 46.3)**	**76**	**37.4 (30.7, 44.2)**	0.337
Encourage the person to contact a helpline	1	0.5 (−0.5, 1.5)	5	2.5 (0.3, 4.7)	7	3.4 (0.9, 6.0)	0.073
Encourage the person to go to hospital	29	14.5 (9.6, 19.4)	30	15.2 (10.1, 20.2)	28	13.8 (9.0, 18.6)	0.928
Encourage the person to go to a mental health hospital	24	12.0^B^ (7.5, 16.5)	49	24.7^A^^C^ (18.7, 30.8)	34	16.7^B^ (11.6, 21.9)	<0.01^m^
Ask if the person wants help	15	7.5 (3.8, 11.2)	11	5.6 (2.3, 8.8)	11	5.4 (2.3, 8.6)	0.625
Assess the problem/risk of harm	15	7.5 (3.8, 11.2)	22	11.1 (6.7, 15.5)	26	12.8 (8.2, 17.4)	0.207
Do an intervention	3	1.5 (−0.2, 3.2)	2	1.0 (−0.4, 2.4)	2	1.0 (−0.4, 2.4)	0.868
Cheer the person up/boost the person's confidence	16	8.0^C^ (4.2, 11.8)	12	6.1^C^ (2.7, 9.4)	30	14.8^A^^B^ (9.9, 19.7)	<0.01^n^
Give advice	14	7.0^B^ (3.4, 10.6)	4	2.0^A^ (0.0, 4.0)	7	3.4 (0.9, 6.0)	<0.05^o^
Seek information for the person	5	2.5 (0.3, 4.7)	5	2.5 (0.3, 4.7)	5	2.5 (0.3, 4.6)	0.999
Help the person make new friends	6	3.0 (0.6, 5.4)	5	2.5 (0.3, 4.7)	11	5.4 (2.3, 8.6)	0.253
Help with chores/work	7	3.5 (0.9, 6.1)	2	1.0 (−0.4, 2.4)	3	1.5 (−0.2, 3.2)	0.167
Provide general support (e.g., practical, emotional)	17	8.5 (4.6, 12.4)	10	5.1 (2.0, 8.1)	14	6.9 (3.4, 10.4)	0.394
Spend time/socialize with the person	11	5.5 (2.3, 8.7)	10	5.1 (2.0, 8.1)	13	6.4 (3.0, 9.8)	0.829
Encourage the person to become physically active	7	3.5 (0.9, 6.1)	12	6.1 (2.7, 9.4)	10	4.9 (1.9, 7.9)	0.490
Tell the person's parents or family	13	6.5 (3.1, 9.9)	16	8.1 (4.3, 11.9)	8	3.9 (1.2, 6.6)	0.219

Symbols flagging table entries denote significant differences relative to

A *GAD*;

B *Schizophrenia*;

C *Depression*.

m *: Schizophrenia vs. GAD P <0.01, OR: 2.41, CI: [1.41, 4.12]*.

*Schizophrenia vs. Depression P <0.05, OR: 1.64, CI: [1.00, 2.67]*.

n *: Depression vs. GAD P < 0.05, OR: 1.99, CI: [1.05, 3.79]*.

*Depression vs. Schizophrenia P < 0.01, OR: 2.69, CI: [1.34, 5.42]*.

o *: GAD vs. Schizophrenia P < 0.05, OR: 3.65, CI: [1.18, 11.29]*.

*The top three most helpful are highlighted in bold*.

Table 7 shows the frequency of participants selecting each outcome as possible scenario for the person described in the vignettes. A majority of the participants endorsed that full recovery would be achieved, but problems would probably recur. A total of 70.7% of the participants for the schizophrenia vignette believed that the person's condition would worsen without professional help, whereas 66.0 and 56.5% held the same belief for the depression and GAD vignettes, respectively.

**Table 7:** Frequency of participants selecting each outcome as a likely scenario for the person described in the vignettes.

**Other interventions**	**GAD vignette**		**Schizophrenia**		**Depression vignette**	
	**(*****n*** **=** **200)**		**vignette (*****n*** **=** **198)**		**(*****n*** **=** **203)**	
	***n***	**%**	***n***	**%**	***n***	**%**
**With professional help**						
Full recovery with no further problems	44	22.0	42	21.2	52	25.6
Full recovery, but problems would probably reoccur	105	52.5	106	53.5	125	61.6
Partial recovery	34	17.0	23	11.6	12	5.9
Partial recovery, but problems would probably reoccur	9	4.5	20	10.1	8	3.9
No improvement	2	1.0	1	0.5	0	0
Get worse	0	0	1	0.5	1	0.5
Don't know	6	3.0	5	2.5	5	2.5
**Without professional help**						
Full recovery with no further problems	5	2.5	2	1.0	7	3.4
Full recovery, but problems would probably reoccur	10	5.0	8	4.0	16	7.9
Partial recovery	12	6.0	8	4.0	10	4.9
Partial recovery, but problems would probably reoccur	10	5.0	14	7.1	10	4.9
No improvement	42	21.0	20	10.1	14	6.9
Get worse	113	56.5	140	70.7	134	66.0
Don't know	8	4.0	6	3.0	12	5.9

## Discussions

This study was the first to use vignettes to investigate the MHL of non-mental health nurses in China. Preliminary data on their identification, attitudes toward treatments, and prognosis for common mental illnesses were presented. Our research has shown that the correct identification rate for the depression vignette was the highest among the three vignettes, and the value was significantly higher than those for the schizophrenia and GAD vignettes. A majority of the participants deemed that the person in each vignette needed professional help, and most of them preferred seeking professional medical help and implementing lifestyle interventions. As for the likely outcomes for the person described in each vignette, more than half of the participants thought that with professional help, the person with the disease would make a full recovery, but problems would probably recur.

### Identification of Disorders in the Vignettes

The correct identification rates for depression and schizophrenia vignettes reached 56.2 and 38.9%, respectively. Less than 20% of the participants correctly labeled the GAD vignette, despite having a list of potential response options. For all the three vignettes, the correct recognition ratios were below 60%, and the correct ratio in each vignette was lower than the corresponding value in our investigation on non-mental health professionals (13). These outcomes are likely due to the role of nurses in the care process; they perform more work on observation, implementing medical orders, and rehabilitation guidance rather than diagnosis. The strong recognition rate for the depression vignette is expected, given the possibility of the high-frequency coverage of the disorder on television and in movies. In the schizophrenia vignette, the secondary diagnosis was “mental illness,” which accounted for 21.2% of the recognition rate. In this vignette, the person acknowledges their mental illness problem but cannot diagnosis it specifically. Although anxiety disorder has become the most prevalent lifetime mental disorder in China (2), the recognition rate in this study was <20%. The low value was due to the people's assumption about anxiety not being a disease, although they consider it as a stress or compulsive disorder. In our research, 15 and 10% of the participants misdiagnosed GAD as stress and obsessive-compulsive disorder, respectively. Therefore, patients with GAD utilize primary care resources frequently, and most of them visit various physicians before they are correctly diagnosed and treated (15). A high level of MHL would increase the possibility of the early identification of these disorders and referral to mental health specialists (13). As one of the important roles in physical treatment, nurses' identification of mental disorders is as important as that of physicians. Serious consequences might be engendered for individuals with mental disorders. Studies have shown that the longer a mental illness is left untreated, the poorer treatment outcomes would be (9, 16, 17). We observed that the longer the nurses work duration was, the higher the recognition rate was for the GAD vignette. This condition may due to the accumulation of experiences with the number of hours worked. In the depression vignette, education level influenced the rate of identification. This finding suggests the importance of enhancing the education level of nurses.

### Helpfulness of Interventions

Most non-mental health nurses deemed that people in the vignettes need professional help. However, several nurses thought that no help was needed, or they were unfamiliar with the correct answer, especially for the GAD vignette. The potential reasons might include the following: the nurses do not consider GAD a disease (18); the disease has not reached the point of requiring treatment. In the schizophrenia vignette, most people thought that the patients needed professional help, probably because psychotic symptoms are perceived as more violent and unpredictable in contrast with those of people suffering from other mental disorders.

As for the people who can help, the results revealed a high rating of perceived helpfulness for treating mental illness by professionals, such as clinical psychologists and psychiatrists, in all the vignettes. The nurses would likely seek professional help. The participants also gave high remarks on seeking help from intimate families and friends. This result accords with the findings by Liu et al. (11) in China and Parker et al. (19) in Singapore. The potential reasons might be the social stigma and perceived discrimination felt by the participants; such conditions and symptoms may be shameful but are considered a private matter in China. Patients prefer handling their mental problems by themselves, and many patients with mental illness opt to conceal their condition. In a previous study carried out in four provinces of China, less than one-fifth of the individuals who met the diagnostic criteria for a mental disorder sought any type of professional help for their condition (20).

At least 60% of the participants in the three vignettes selected correctly the medications that could help. The rate was higher than the identification rate, possibly because in this question, participants can select more than one option and would opt for all the possible right answers. This finding may reflect the participants' ability to identify the disease. Such belief can positively affect treatment outcomes. However, more than 30% of the participants thought that vitamins, minerals, and herbal medicines were helpful in all the vignettes, and more than 10% endorsed laxatives and antibiotics as effective medications. Affected by Chinese traditional culture, many people believe that Chinese medicine is better than Western medicine and features less side effects.

Our results showed that 80.5, 82.8, and 84.3% of participants labeled “getting out more” as helpful for the GAD, depression, and schizophrenia vignettes, respectively. The results show that the participants lack knowledge about mental illness and understanding of the importance of early treatment. For the schizophrenia vignette, the other common helpful choices were being admitted to a psychiatric ward of a general or a psychiatric hospital, followed by being physically more active. Views on electroconvulsive therapy (ECT) were negative for the three vignettes, especially those with GAD and depression. However, ECT is a safe and efficacious treatment for specific mental illnesses. The medical staffs' negative views on ECT may prompt patients and their families to refuse this procedure when necessary. Therefore, the necessary efforts for educating these professionals and exploring the possibility of brief interventions must be considered in future studies.

### Best Methods of Help

The rate for listening/talking with the person was the highest among all the help methods for the GAD and depression vignettes. Although this option was not the first for the schizophrenia vignette, it was followed by encouraging the person to consult a psychologist. This result was consistent with the above questions about interventions, suggesting that the participants believed that help from families or friends should be considered first, especially for the GAD and depression vignettes. Such result is expected given the low identification rates for the three vignettes, that is, people failed to recognize these conditions as diseases. People's identification of disease influences their choices of treatment. Thus, seeking help from professionals is not consequently considered necessary. Turning to family and friends for help causes no problems if they provide appropriate support, or if professional help is also sought (21).

### Beliefs About Outcomes

Most participants expressed the same opinion on the prognosis of the three vignettes with or without professional treatment. The results show promise because more than half of the participants believed that the person with the diseases will fully recover with professional help, but problems would probably recur and worsen without professional help. However, the correct choice was the lowest for the GAD vignette, consistent with the identification rate. Numerous mental illnesses are chronic and persistent, with several exhibiting spontaneous remissions. This result may reflect that certain people, particularly GAD patients, would seek other non-professional interventions.

Our research showed the inadequate MHL of non-mental health nurses in China, especially the MHL for GAD. First, the identification rate was low, and the highest recognition rate was observed for the depression vignette. Although most participants for the GAD vignette thought that people with mental disorders need professional help, several deemed that help is unnecessary. In terms of specific interventions, more than 60% of the participants selected the correct drug, but several preferred giving higher evaluation scores to vitamins, minerals, and herbal medicines. Most participants thought that intervention from professional medical staff was helpful, and they also gave high ratings on lifestyle interventions. Notably, the ECT was rated with relatively low scores. More than 50% of the participants provided the right choice in the prognosis of mental disorders. These results could partially explain why many individuals with mental disorders never access services in their whole life (20). Given that the participants in our research completed a secondary specialized degree or above, and a series of research showed that increased health literacy is associated with better recognition of health problem, that is, the public may possess lower MHL (22–24). People with mental illness experience increased medical comorbidities and feature higher mortality rates than the general population (25). They may first seek treatment for somatic problems in non-mental health settings, or they may be transferred to non-mental health settings. Such condition should alert nurses and other healthcare professionals to better understand and improve mental health care for this group. Efforts should be taken to increase the medical personnel's MHL.

To close the gap in access to mental health services worldwide, the World Health Organization highlighted in its Comprehensive Mental Health Action Plan 2013–2020 an objective for all countries to achieve by 2020 (26): “to provide comprehensive, integrated and responsive mental health and social care services in community-based settings.” China has exerted efforts to achieve this goal. However, several proposals have yet to be fully activated, particularly those that focus on integrated care, thus leaving the country with serious challenges. For common mental disorders, the rates of contact (i.e., patients having contact with any general or mental healthcare providers) and provision of mental health services (i.e., delivery of evidence-based interventions, patient adherence to treatment, and retention of care and clinical and social outcomes) are low (27). As we mentioned before, mental health services and professionals are scarce in China. In addition to cultivating qualified people as soon as possible, we must find other possible ways to fill the lack of resources in this area. Increasing knowledge and positive attitudes about mental illness among the general population may improve the extent to which individuals seek help for and disclose a mental illness (28). Given that nurses are the persons who are in close contact with patients and their families, their beliefs about mental illnesses are crucial not only in providing appropriate and prompt referrals but also in influencing their attitudes toward people with mental illness. Improving the MHL of nurses can improve effective nursing interventions which may be critical to address the special needs of people with mental illness. In the absence of specialized education programs, nurses cannot provide adequate help for patients. Supplementing the education and training about mental health among nurses in China may be helpful. Actions should be taken to provide training programs to improve knowledge about mental health, awareness of how to seek help and treatment, and reduce the stigma against mental illness at the individual, community, and institutional levels. Such actions may promote the early identification of mental disorders, improve mental health outcomes, and increase the use of health services.

## Conclusions

In conclusion, the MHL of non-mental health3 nurses in China is insufficient. This condition is particularly true for the case for GAD. Their ability to identify common mental diseases and their beliefs in helpful intervention and outcome need to be enhanced to reduce the disease burden caused by mental illness. More programs are needed to improve the MHL of non-mental health nurses.

### Limitation

This study features major limitations that must be acknowledged. First, we only investigated four hospitals from one region, which might lead to a risk of bias in a relatively homogeneous sample. Second, the sample size was relatively small and may not represent the average MHL level of all non-mental health nurses in China. The current preliminary study must be extended to use a new and larger sample in the future research. Third, our research is a cross-sectional study that excluded determining an exact causal relationship. We have no way to assess how the nurses' MHL affects referrals or treatments of patients. Moreover, we listed multiple options for diagnosis and interventions, which might restrict the participants' choice and thoughts. We also only assessed their beliefs about interventions and not their practice in actual work. Furthermore, the professionals' behavior might be affected by local contextual factors such as accessibility to resources.

## Data Availability Statement

The datasets generated for this study are available on request to the corresponding author.

## Ethics Statement

The research program was approved by the ethics committee of the Second Xiangya Hospital of Central South University. Before completing the questionnaires, we stated the aim of the study and requested consent from the participants to participate by filling in the questionnaire.

## Author Contributions

TL was responsible for the study design, manuscript preparation, and revision. YH performed statistical analysis and wrote the first draft of the manuscript. QW wrote the protocol and revised the manuscript drafts. QW, XL, SC, CQ, and YX were responsible for data collection. All authors have contributed to and have approved the final manuscript.

## Conflict of Interest

The authors declare that the research was conducted in the absence of any commercial or financial relationships that could be construed as a potential conflict of interest.

## Abbreviations

ECT: electronic therapy
GAD: generalized anxiety disorder
MHL: mental health literacy
PTSD: post-traumatic stress disorder.
